# Persistent p55TNFR expression impairs T cell responses during chronic tuberculosis and promotes reactivation

**DOI:** 10.1038/srep39499

**Published:** 2016-12-20

**Authors:** Ivy M. Dambuza, Roanne Keeton, Nai-Jen Hsu, Nasiema Allie, Valérie F. J. Quesniaux, Bernhard Ryffel, Muazzam Jacobs

**Affiliations:** 1 Division of Immunology, Department of Pathology and Institute of Infectious Disease and Molecular Medicine, Faculty of Health Sciences, University of Cape Town, South Africa; 2 CNRS UMR7355, Experimental and Molecular Immunology and Neurogenetics, 45071 Orleans, France; 3 National Health Laboratory Service, South Africa; 4 South African Medical Research Council, Cape Town, South Africa.

## Abstract

The pleiotropic activities of TNF are mediated by two structurally related but functionally distinct type I transmembrane receptors, p55TNFR and p75TNFR expressed in most cell types, that can be cleaved and act as TNF scavengers. Here, we investigated the effect of persistent p55TNFR cell surface expression during aerosol inhalation challenge with virulent *M. tuberculosis* H37Rv. We demonstrated that persistency of p55TNFR in macrophage cultures increased the synthesis of soluble TNF, p75TNFR and NO, however, had no effects on bacteria killing ability. Furthermore, it did not facilitate enhanced protection to primary acute *M. tuberculosis* infection in p55^∆NS^ mice. Without exacerbated lung inflammation, we found a compensatory increase in p75TNFR shedding and decrease in bioactive TNF in BAL of p55^∆NS^ mice after *M. tuberculosis* challenge. Defective expressions of CD44 and INFγ attributed to an impaired T cell response during persistent p55TNFR expression that caused marginal transient susceptibility during chronic infection. Moreover, persistent p55TNFR expression induced early reactivation during latent tuberculosis infection. These data indicate a prominent role of p55TNFR shedding in Th1 mediated protection against chronic and latent tuberculosis infection.

Tumor Necrosis Factor (TNF) is a member of the TNF superfamily (TNFSF), a class of structurally related cytokines that are involved in diverse immunological and developmental pathways^1^,^2^. Murine TNF is initially synthesized as a 26 kDa glycosylated type II transmembrane molecule (Tm-TNF) that can be released from the cell surface by the metalloprotease TNF-α-converting enzyme (TACE) to generate a 17 kDa protein, and subsequent to homotrimerisation, a 51 kDa soluble TNF (solTNF) molecule is formed^3^,^4^,^5^,^6^. Both TNF forms are biologically active and their extensive biological effects are mediated by two receptors: p55TNFR (TNFRSF1A, CD120a, TNFR1) and p75TNFR (TNFRSF1B, CD120b, TNFR2) with Tm-TNF preferentially signaling through p75TNFR and solTNF binding strongly to p55TNFR^7^. Both receptors are type I transmembrane proteins sharing approximately 25% homology in their extracellular domains but have divergent cytoplasmic domains. Their extracellular domains can be released from the cell surface through proteolysis mediated by metalloproteases of the ADAM family, thereby generating soluble TNFRs capable of binding TNF^8^,^9^. Soluble TNFRs are thought to serve as (a) TNF antagonists, (b) TNF carrier proteins (c) a slow release reservoir for TNF, and as (d) stabilizers of TNF-bioactivity^10^ and have been detected during various disease states including typhoid fever^11^, malaria^12^, sub-acute bacterial endocarditis^13^ and visceral leishmaniasis^14^, and mycobacterial infections including tuberculosis^15^,^16^,^17^. Numerous clinical studies have demonstrated increased serum TNF receptors concentrations, associated with severity of M. tuberculosis disease^18^,^19^. TNF shares its receptors with Lymphotoxin (LT); in its homotrimeric form LTα interacts with TNFRp55 and p75 while the LTαLTβ heterotrimers, interact with LTβ-R^20^.

TNF gene deletion^21^,^22^,^23^,^24^, p55TNFR gene deletion^16^,^25^,^26^, and TNF neutralization studies^27^,^28^ demonstrated TNF-TNFR signaling as critical for protective immunity against mycobacterial infections. In the absence of TNF-p55TNFR signaling, challenge of mutant mice with either attenuated *M. bovis* BCG^29^ or virulent *M. tuberculosis*^21^,^24^,^25^ resulted in elevated bacterial burdens, impaired granuloma structure formation and early death. Owing to its strong pro-inflammatory and immunostimulatory activities, the duration and amplitude of TNF functions are tightly regulated. One mechanism, which controls bioavailability of TNF, is p55TNFR and p75TNFR shedding. Both soluble p55TNFR and p75TNFR are found in the lung of *M. tuberculosis* infected patients and mice^15^,^16^,^30^,^31^. However, virulent pathogens such as *M. tuberculosis* H37Rv and *M. avium* can actively manipulate this system to evade the immune response^15^,^16^,^32^. Indeed, we showed recently that a major role of p75TNFR in mycobacterial infection control seems to be through its shed soluble form, which effectively regulate the level of bioactive TNF that contributes to the disease outcome^16^. Patients treated with TNF inhibitors including Etanercept, a p75TNFR IgG fusion protein show a marked increased risk for TB infection^33^.

Here we address the specific role of shed, soluble p55TNFR in host control of M. tuberculosis infection. The generation of a nonsheddable p55TNFR (p55^ΔNS^) mouse strain has provided insight into the roles of both the soluble and the membrane-bound forms of p55TNFR in pathogenesis of infectious, inflammatory and autoimmune diseases. Xanthoulea and colleagues indicated that mice expressing a mutated nonsheddable form of p55TNFR (p55^∆NS^) developed a Toll-like receptor dependent innate immune hyper responsiveness^34^. In this mouse model p55TNFR is rendered nonsheddable on all cell types which express p55TNFR. We thus investigated the role of signaling and regulatory effects of nonsheddable p55TNFR in host immunity against *M. tuberculosis*. We report here after *M. tuberculosis* aerosol exposure that p55^∆NS^ mice expressing nonsheddable p55TNFR controlled acute infection similar to their WT counterparts while they exhibited a transient loss of control during chronic infection and an increased susceptibility to reactivation of latent tuberculosis.

## Results

### Sustained p55TNFR surface expression in p55^∆NS^ peritoneal elicited cells after *M. tuberculosis* infection

**Figure 1:**
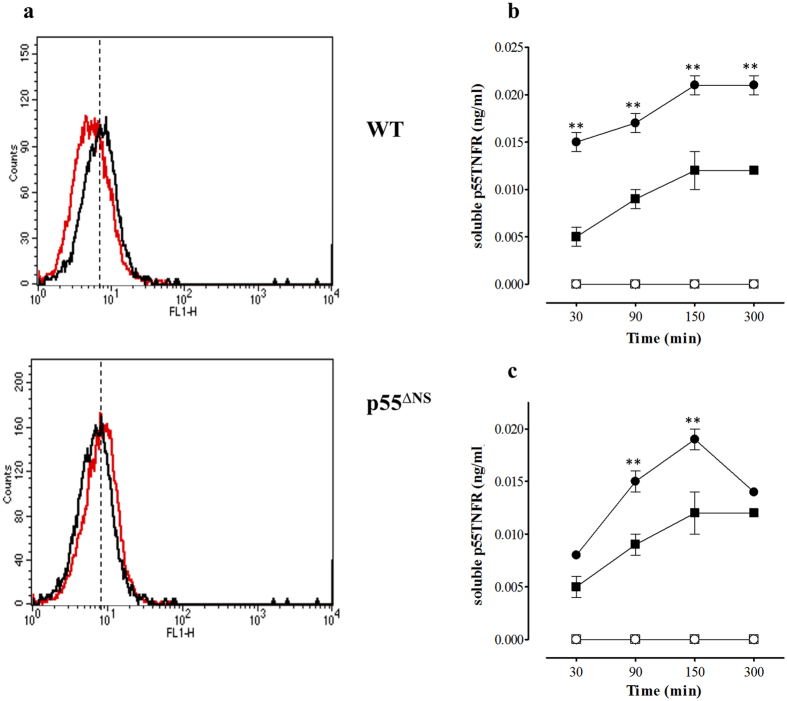
Sustained p55TNFR cell surface expression in p55^∆NS^ cells. Thioglycollate elicited peritoneal macrophages from p55^∆NS^ and WT mice were stained with anti-p55TNFR before stimulation (black histogram) or after 20 minutes stimulation with *M. tuberculosis* H37Rv (MOI = 2:1) and analyzed by flow cytometry to determine p55TNFR cell surface expression (**a**) (red histogram, MFI loss indicates receptor shedding). Activation induced soluble p55TNFR cleavage was observed in WT but not p55^∆NS^ cells. Thioglycollate elicited peritoneal macrophages were isolated from WT mice (closed squares represent resting cells and closed circles represent stimulated cells) and p55^∆NS^ mice (open squares represent resting cells and open circles show stimulated cells) and stimulated with 100 ng/ml LPS (**b**) or *M. tuberculosis* H37Rv (MOI = 2:1) (**c**). Soluble p55TNFR accumulation in the supernatants was determined by ELISA at time points indicated. Data are expressed as mean ± SD triplicate values and results represent 1 of 2 experiments performed. Statistical analysis compared resting and stimulated macrophages and was performed by ANOVA (*p < 0.05, **p < 0.01).

Numerous studies have demonstrated release of TNFRs after infection or immunization *in vitro* and *in vivo*^15^,^16^. TNFR shedding from cell surfaces coincides with the generation of TNF^16^,^17^; its functional significance being associated with the regulation of TNF mediated effects^35^,^36^,^37^,^38^. Xanthoulea and colleagues showed that upon activation with PMA, peritoneal exudate cells derived from p55^∆NS^ mice expressed a nonsheddable p55TNFR^34^. Here, we investigated the relationship between p55TNFR expression and *M. tuberculosis* infection. Thioglycollate elicited macrophages from WT mice or p55^∆NS^ mice were exposed to *M. tuberculosis* H37Rv for 20 minutes and p55TNFR cell surface expression determined by flow cytometry. p55TNFR expression occurred in both WT and p55^∆NS^ unstimulated macrophages (Fig. 1a). However, exposure to *M. tuberculosis* H37Rv caused a loss of p55TNFR surface expression on WT macrophages (reflected by reduced Mean Fluorescent Intensity) compared to the elevated and sustained expression noted in p55^∆NS^ macrophages (Fig. 1a). Quantification of the cleaved p55TNFR revealed detectable levels of soluble p55TNFR present in unstimulated culture supernatants of thioglycollate elicited macrophages from WT mice, which increased significantly at all the time points (p < 0.01) after activation with LPS (Fig. 1b) and increased significantly at 90 and 150 minutes after viable *M. tuberculosis* H37Rv (Fig. 1c) infection (p < 0.01). No soluble p55TNFR was detected in supernatants of p55^∆NS^ macrophages before or after stimulation.

The data confirms sustained expression of p55TNFR in p55^∆NS^ mice and demonstrate that p55TNFR shedding is induced by *M. tuberculosis*.

### Enhanced macrophage bactericidal potential does not correlate with increased bacilli killing in the presence of sustained p55TNFR expression

**Figure 2:**
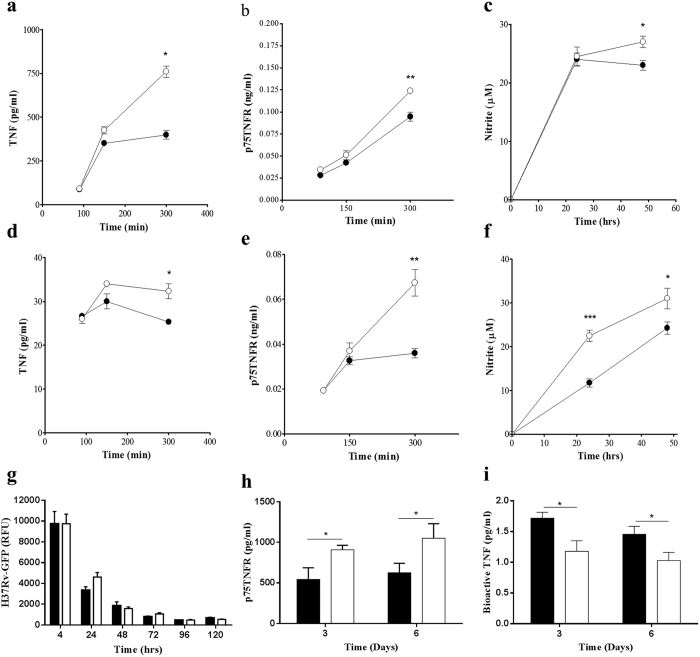
Enhanced TNF, Nitric Oxide and TNFRp75 production in macrophages expressing a non-sheddable form of p55TNFR. Bone marrow derived macrophages isolated from WT mice (closed circles) and p55^∆NS^ mice (open circles) were prestimulated with 100 U/ml IFNγ for 24 h prior stimulation with 100 ng/ml LPS (**a**–**c**) or *M. tuberculosis* H37Rv (**d**–**f**), (MOI = 2:1). The kinetics of soluble TNF (**a**,**d**) and TNFRp75 (**b**,**e**) accumulation in the supernatants was measured by ELISA, and the production of NO was quantified by measuring the nitrite concentration using Griess reagent (**c**,**f**). Macrophage bactericidal capacity to kill *M. tuberculosis* bacilli was analysed by measuring relative fluorescent unit (RFU) in H37Rv-GFP infected macrophages using the GFP-microplate assay (**g**). The levels of soluble p75TNFR (**h**) and bioactive TNF (**i**) were measured in the BAL fluids of WT (closed bars) and p55^∆NS^ mice (open bars) at 3 and 6 days after *M. tuberculosis* infection. Results represent 1 of 3 similar experiments and data are expressed as mean ± SD of triplicate values. Statistical analysis was performed by ANOVA (*p < 0.05, **p < 0.01, ***p < 0.001).

We hypothesized that sustained p55TNFR expression may allow for continued or amplification of TNF signalling yielding improved control of infection. To determine how sustained membrane p55TNFR expression influences restriction of bacterial replication, we investigated the bactericidal potential and bacterial killing in bone marrow derived macrophages (BMDM). Unlike the thioglycolate elicited macrophages, BMDM primary cultures represent a more naïve homogeneous macrophage population which can be stimulated and activated under different experimental conditions. These BMDM’s from WT mice and p55^∆NS^ mice were pre-activated with (equimolar) 100 U/mL IFNγ concentrations for 24 hours to achieve optimal macrophage activities similar to those during inflammation, in particular the NO production by active macrophages. This requirement for IFNγ pre-activation was confirmed by initial optimization studies in which nitrite production were below the baseline without IFNγ pre-stimulation (Supplementary Fig. 1). TNF and nitric oxide (NO) levels were quantified following stimulation with LPS or *M. tuberculosis* H37Rv. Consistent with published data^34^, stimulating p55^∆NS^ derived macrophages with 100 ng/ml LPS increased TNF synthesis in a time-dependent manner with a significant increase (*p* < 0.05) observed 300 minutes post stimulation compared to WT derived macrophages (Fig. 2a). Similarly, significantly higher TNF production was observed in p55^∆NS^ derived macrophages when challenged with *M. tuberculosis* H37Rv, however TNF concentrations were found to be approximately 10 fold less compared to LPS stimulation (Fig. 2d).

TNF and IFNγ synergize for optimum macrophage activation resulting in production of the effector molecule NO^39^,^40^, which can be measured relatively by the Griess Reagent System to determine the nitrite (NO_2_^−^) concentration as one of two primary stable breakdown products of NO. Therefore, we quantified nitrite concentrations in supernatants of activated macrophages and found levels significantly enhanced (*p* < 0.05) in p55^∆NS^ derived macrophages relative to WT controls after stimulation with LPS for 48 hours (Fig. 2c). Similar results were attained when macrophages were stimulated with *M. tuberculosis* H37Rv, with a significant increase in nitrite concentration (*p* < 0.001) as early as 24 hours after stimulation and a further increase in NO synthesis (*p* < 0.05) observed 48 hours later in p55^∆NS^ mice compared to WT mice (Fig. 2f). Therefore, the increased synthesis of TNF and NO in p55^∆NS^ macrophages points to a heightened state of macrophage activation and bactericidal potential. On the contrary, *M. tuberculosis*-GFP bacilli killing assays revealed no significant enhancement of bactericidal ability in p55^∆NS^ macrophages compared to WT macrophages (Fig. 2g).

Both TNFRs are proteolytically cleaved from the cell surface in the presence of inflammatory stimuli and the levels of soluble TNFRs are thought to modulate TNF responsiveness. We and others have shown that increased shedding implied a more dominant role for p75TNFR in regulating the bioactivity of TNF^16^,^41^. To determine whether there was a difference in p75TNFR shedding between WT and p55^ΔNS^ derived macrophages, the levels of soluble p75TNFR were also quantified in the supernatants after stimulation with LPS or *M. tuberculosis* H37Rv. Results showed that p55^ΔNS^ derived macrophages produced significantly higher levels (*p* < 0.01) of soluble p75TNFR when stimulated with either LPS (Fig. 2b) or *M. tuberculosis* H37Rv (Fig. 2e) for 300 minutes. To ascertain whether p75TNFR shedding affects the bioactivity of TNF in p55^ΔNS^ mice, we challenged the mice with M. tuberculosis H37Rv by aerosol inhalation. Here we found higher levels of p75TNFR and lower levels of bioactive TNF in the bronchoalveolar lavage (BAL) fluid of p55^ΔNS^ mice compared to WT mice at both day 3 and day 6 post-infection (Fig. 2h,i) that corroborates our previous findings^16^. These data suggest that defective p55TNFR shedding results in significant increased release of soluble p75TNFR as a compensatory mechanism with potential to modulate TNF activity.

### Sustained expression of surface p55TNFR does not enhance mycobactericidal responses during acute *in vivo M. tuberculosis* infection

**Figure 3:**
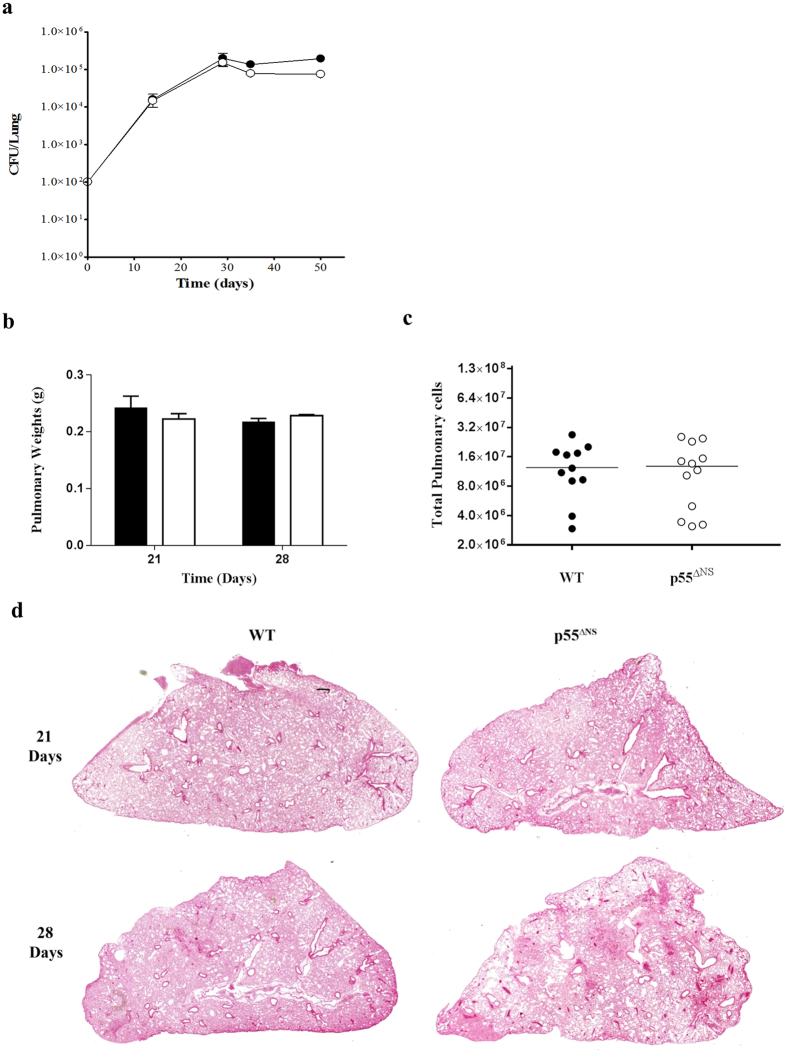
Persistent membrane p55TNFR expression does not enhance control of mycobacterial replication during acute infection. WT mice (closed circles) and p55^∆NS^ mice (open circles) were challenged by aerosol inhalation with 50–100 CFUs/mouse with *M. tuberculosis* H37Rv. Bacilli burdens were enumerated in the lungs (**a**) at time points indicated above. Lung weights (**b**) were recorded at 21 and 28 days post-infection as a surrogate marker of infllamation. Total pulmonary cell numbers (**c**) were determined at 21 days. Histopathology was assessed by (**h**,**e**) staining of formalin-fixed lung (**d**). Data are representative of 3 similar experiments. Results are expressed as mean ± SD of 4 animals/group. Statistical analysis was performed by ANOVA (*p < 0.05, **p < 0.01, ***p < 0.001).

The abrogation of TNF-p55TNFR signaling, either genetically or with neutralizing antibodies, results in susceptibility to mycobacterial and other intracellular infections^21^,^25^,^28^,^42^. Specific functions attributable to soluble and membrane TNFRs in various infections and disease states are still not fully understood. In both mice and humans, defective p55TNFR shedding resulted in enhanced sensitivity to TNF mediated immunopathology^43^,^44^. Xanthoulea and colleagues reported enhanced innate immune activation in p55^∆NS^ mice, which mediated increased resistance to *L. monocytogenes* infection *in vivo* and suggested that persistently expressed membrane p55TNFR conferred enhanced protective antibacterial immunity^34^. In consideration of these results, we addressed the *in vivo* functional role of membrane bound p55TNFR and receptor shedding during *M. tuberculosis* H37Rv infection. WT mice and p55^∆NS^ mice were exposed to a low dose aerosol inhalation infection of 50–100 CFUs/mouse *M. tuberculosis* H37Rv and pulmonary bacilli burdens were assessed over 8 weeks. Results showed that p55^ΔNS^ mice controlled acute infection with comparable kinetics to WT mice reflected by equivalent bacilli numbers in the lungs (Fig. 3a) at all the acute time points investigated. We also observed similar degrees of pulmonary inflammation in terms of comparable lung weights (Fig. 3b) and total cell counts of the lung (Fig. 3c). Further, pulmonary pathology was qualitatively equivalent with no differences in cellular infiltration or granuloma formation (Fig. 3d). Thus sustained p55TNFR expression does not enhance the bactericidal capacity of the host during acute *M. tuberculosis* infection.

### Macrophage function is not enhanced in the presence of sustained p55TNFR expression *in vivo* during acute *M. tuberculosis* infection

**Figure 4:**
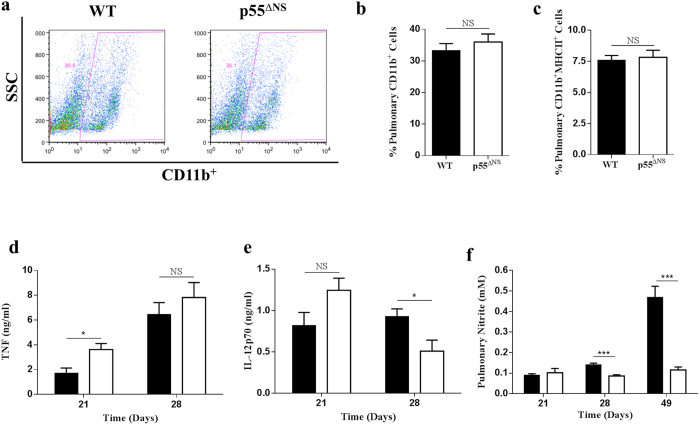
Similar expression of CD11b^+^/MHC-II^+^ cells and increased TNF and IL-12p70 expression in the lungs of M. tuberculosis infected p55^∆NS^ mice. WT mice (closed bars) and p55^∆NS^ mice (open bars) were infected by aerosol inhalation with 50–100 CFUs/mouse M. tuberculosis H37Rv. Single cell suspensions were generated from PBS-Heparin perfused lungs at day 21 post-infection, stained and analyzed for CD11b and MHC-II expression by flow cytometry. Representative dot plots representing flow cytometry analysis of CD11b^+^ cells (**a**) obtained from the infected lungs of WT mice. Percentages of pulmonary CD11b^+^ (**b**) and CD11b^+^/MHC-II^+^ (**c**) cells are presented. For cytokine expression, lungs were isolated and homogenized at days 21 and 28 post-infection and the levels of TNF (**d**) and IL-12p70 (**e**) were quantified by ELISA. The production of NO was quantified by measuring the nitrite concentration using Griess reagent (**f**). Data represent 1 of 3 experiments performed and results are expressed as mean ± SD from 4 mice/group. Statistical analysis was performed by ANOVA (*p < 0.05, **p < 0.01, ***p < 0.001).

To understand how persistent p55TNFR expression affects macrophage function *in vivo* we measured and compared recruitment of CD11b^+^ cells to the lungs and their activation state in *M. tuberculosis*-infected WT mice and p55^ΔNS^ mice. Flow cytometric analyses of pulmonary cells revealed equivalent frequencies of CD11b^+^ cell recruitment in p55^∆NS^ mice compared to WT mice at day 21 post-infection (Fig. 4a,b; Gating strategy in Supplementary Fig. 2). To determine macrophage activation under conditions of sustained p55TNFR presence, we analyzed the frequencies of CD11b^+^MHC-II^+^ cells as well as the MFIs of cell surface MHC-II, CD80 and CD86 expression on CD11b^+^ cells in the lungs at 21 days post-infection. p55^ΔNS^ mice displayed comparable CD11b^+^MHC-II^+^ cells compared to WT mice 21 days post infection (Fig. 4c). The number of CD11b^+^CD80^+^ and CD11^+^CD86^+^ cells as well as the MFIs of MHC-II, CD80 and CD86 expressed on CD11b^+^ cells was also found to be equivalent in both strains (Data not shown). The data indicate that persistent membrane p55TNFR expression does not enhance the expression of costimulatory molecules on CD11b^+^ cells.

To determine the influence of persistent p55TNFR expression on macrophage function *in vivo*, IL-12p70, NO and TNF concentrations were measured in the lungs of *M. tuberculosis*-infected WT mice and p55^∆NS^ mice. IL-12p70 and TNF are critical mediators of anti-mycobacterial immunity and primarily synthesized by macrophages^45^,^46^,^47^,^48^,^49^. TNF levels in p55^∆NS^ mice were significantly higher (*p* < 0.05) at day 21 compared to WT mice (Fig. 4d), while IL-12p70 concentrations showed a non-significant trend towards enhancement in p55^∆NS^ mice compared to WT mice at 21 days post-infection but was significantly reduced si at day 28 (Fig. 4e). NO levels in lung homogenates of p55^∆NS^ mice were significantly lower (p < 0.001) compared to WT mice at 28 and 49 days post-infection (Fig. 4f). Collectively the data indicates that sustained p55TNFR expression does not enhance macrophage activation.

### Th1 immune activation is impaired in p55^∆NS^ mice

**Figure 5:**
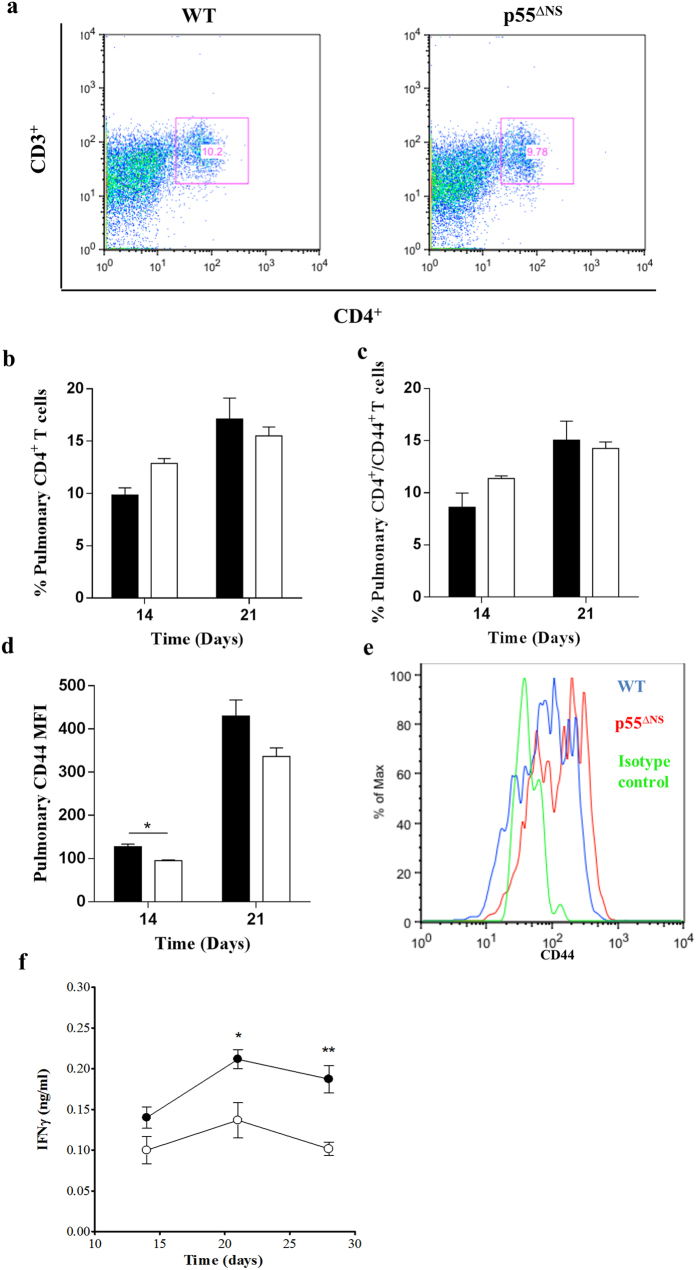
T cell responses in the presence of persistent membrane p55TNFR expression. WT mice (closed circles/bars) and p55^∆NS^ mice (open circles/bars) were infected by aerosol inhalation with 50–100 CFUs *M. tuberculosis* H37Rv. Representative dot plots of flow cytometry analysis showing CD4^+^CD44^+^ cells (**a**) obtained from the infected lungs of WT mice. Pulmonary single cells suspensions from PBS-Heparin perfused lungs were analyzed for CD4^+^ (**b**) and CD4^+^CD44^+^ (**c**) expression, and the CD44 MFI (**d** and representative from day 14 in (**e**): IgG control – green, WT – red, p55^∆NS^ - blue) calculated. For pulmonary cytokine expression, IFNγ (**f**) synthesis was quantified by ELISA. Results represent 1 of 2 experiments performed and data are expressed as mean ± SD of 4 mice/group. Statistical analysis was performed by ANOVA (*p < 0.05, **p < 0.01, ***p < 0.001).

As a key determinant of disease outcome we investigated whether sustained p55TNFR expression influences Th1 immune function. Here, we first evaluated the influx and activation state of CD4^+^ T-cells in the lungs of infected WT mice and p55^∆NS^ mice by flow cytometry. CD44 and IFNγ were used as markers of T cell activation. CD44 is a cell adhesion receptor and wildly distributed. Ligation of CD44 with hyaluronanis results in a variety of downstream responses that includes cell adhesion, cell migration, and hematopoietic process. In mature lymphocytes, CD44 is upregulated in response to antigenic stimuli and represents the effector stage of immunological responses^50^,^51^,^52^. The use of CD44 as marker for effector T cells is generally accepted and demonstrated in various fields of studies^16^,^53^,^54^. Comparable frequencies of CD4^+^ T cells were present between the two strains at both day 14 and day 21 post-infection (Fig. 5a,b; Gating strategy in Supplementary Fig. 2), Interestingly while equivalent frequencies of pulmonary CD4^+^CD44^+^ T cells were measured in both strains 14 days and 21 days post-infection (Fig. 5c), the MFI of cell surface CD44 expression gated on CD4^+^ T cells was lower in p55^∆NS^ mice compared to WT mice, significantly so (*p* < 0.05) at day 14 post-infection (Fig. 5d,e). The data illustrate that despite the comparable CD4^+^ T cell recruitment to lungs of WT mice and p55^∆NS^ mice, persistent p55TNFR expression might play a suppressive role in T cell activation during early infection.

We next compared the kinetics of IFNγ production in the lungs of infected WT mice and p55^∆NS^ mice as a further measurement of *in vivo* T cell functionality. We observed lower IFNγ concentrations with significant differences at day 21 (*p* < 0.05) and day 28 (*p* < 0.01) post-infection in p55^∆NS^ mice compared to WT mice (Fig. 5f). The data suggest that persistent p55TNFR expression may suppress pulmonary T cell activation which associates with reduced IFNγ production.

### Sustained expression of surface p55TNFR results in marginally impaired bactericidal responses during chronic *in vivo M. tuberculosis* infection

**Figure 6:**
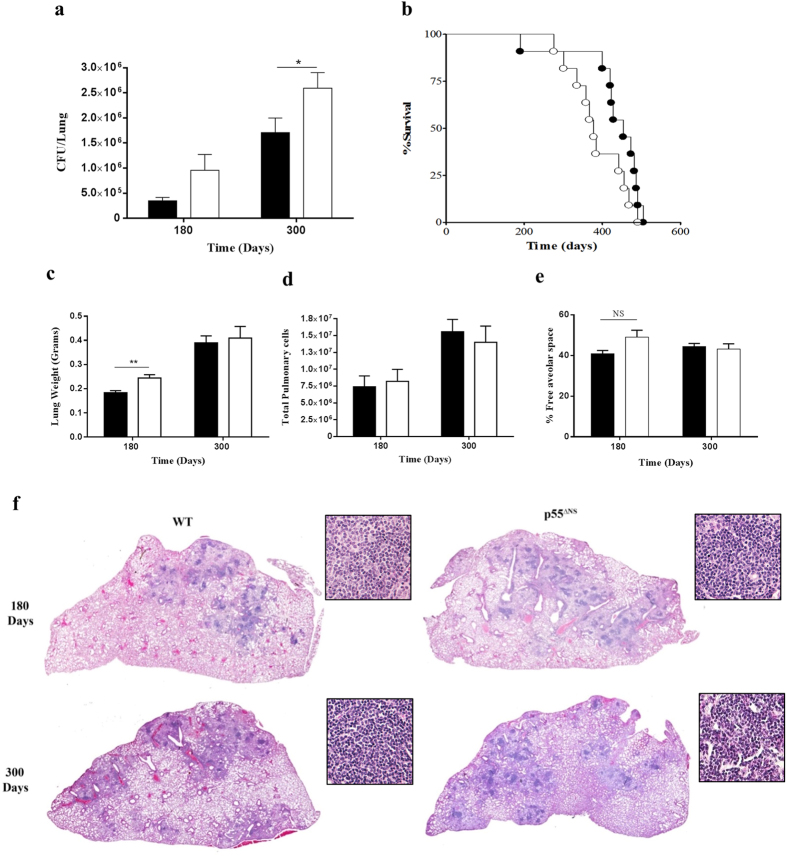
Persistent membrane p55TNFR expression causes in increased bacterial burden and increased sensitivity during chronic infection. WT mice (closed circles) and p55^∆NS^ mice (open circles) were challenged by aerosol inhalation with 50–100 CFUs/mouse with *M. tuberculosis* H37Rv. Bacilli burdens were enumerated in the lungs (**a**) at time points indicated above. Mortality rates were monitored over the indicated infection period (n = 11) (**b**). At 180 and 300 days post-infection lung weights were recorded as a surrogate marker of inflammation (**c**) and total pulmonary cell counts determined (**d**). Histopathology was assessed by (**h**,**e**) staining of formalin-fixed lungs (**f**) and subsequent analysis of free alveolar space (**e**). Additional magnified images (200X) of the granulomatous regions were also included in (**e**). Data are representative of 2 similar experiments. Results are expressed as mean ± SD of 4 animals/group. Statistical analysis was performed by ANOVA (**a**,**c**,**d**,**e**) or Logrank (**b**) (*p < 0.05, **p < 0.01, ***p < 0.001).

We postulated that a dysregulated Th1 immune response in p55^∆NS^ mice may compromise long term host protective immunity during a primary infection. Therefore, to determine the effect of sustained p55TNFR expression on disease outcome during chronic stages of a primary infection, *M. tuberculosis* infected p55^∆NS^ mice and WT mice were monitored for >500 days. The bacilli burdens of p55^∆NS^ mice were higher than those of WT mice at 180 days post-infection and, although moderate, was nonetheless significantly lower at 300 days post-infection (p < 0.05) (Fig. 6a) which correlated with a non-significant trend towards more rapid death in p55^∆NS^ mice as compared to WT controls, where WT mice had an increased medium survival of 476 days compared to 395 days in p55^∆NS^ mice (Fig. 6b). Although the analysis of total lung cell counts (Fig. 6d) and free alveolar space (Fig. 6e) showed no statistical difference, we observed a transient increase in lung histopathology (Fig. 6f) at 180 days, which was quantitatively confirmed by an increase in lung weights (Fig. 6c) at this time point in p55^∆NS^ mice.

The data therefore indicates that persistent p55TNFR surface expression compromises protection against chronic *M. tuberculosis* infection.

### Sustained expression of surface p55TNFR results in loss of control against reactivation of latent mycobacterial infection

**Figure 7:**
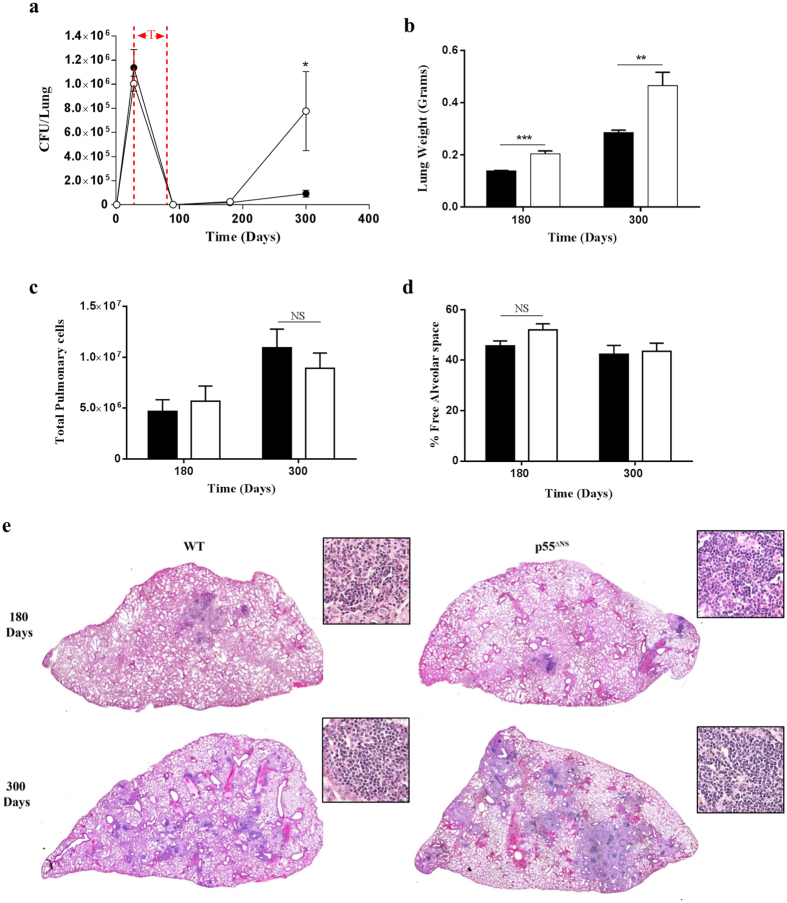
Persistent membrane p55TNFR expression induces loss of control during latent M. tuberculosis infection. WT mice (closed circles/bars) and p55^∆NS^ mice (open circles/bars) were challenged by aerosol inhalation with 50–100 CFUs/mouse with *M. tuberculosis* H37Rv. Latency was achieved by treatment with RIF/INH (25 mg/kg) for 6 weeks commencing at day 28 post-infection (Red lines marked as T in **a**). Bacilli burdens were enumerated in the lungs (**a**) at time points indicated above. At 180 and 300 days post-infection lung weights were recorded as a surrogate marker of inflammation (**b**) and total pulmonary cell counts analyzed (**c**). Histopathology was assessed by (**h**,**e**) staining of formalin-fixed lungs (**e**) and subsequent analysis of free alveolar space (**d**). Additional magnified images (200X) of the granulomatous regions were also included in (**e**). Data are representative of 2 similar experiments. Results are expressed as mean ± SD of a minimum of 4 animals/group. Statistical analysis was performed by ANOVA (*p < 0.05, **p < 0.01, ***p < 0.001).

Our data demonstrated that persistent p55TNFR expression might play a suppressive role in T cell activation, we next assessed the quality of the T cell response generated in a reactivation challenge model. The rationale was that defects in the development of a fully functional T cell response during the primary infection might compromise the ability of *M. tuberculosis* specific T cells to protect against reactivation of a latent infection. Infected p55^∆NS^ mice and WT mice were treated with rifampicin and isoniazid for 6 weeks, beginning at day 28 post-infection to induce a state of latency and were subsequently assessed at 90, 180, 300 days for reactivation of tuberculosis. Similar to our earlier finding, the acute phase of latent infection showed no differences in CFU burdens (Fig. 7a). Low bacilli burdens measured at 90 days post-infection confirmed successful chemotherapy in both strains. However p55^∆NS^ mice showed enhanced reactivation of disease with significantly higher bacilli burdens by 300 days (Fig. 7a) accompanied by significantly higher lung weights (Fig. 7b) compared to WT control mice. Despite the equivalent numbers of total lung cells (Fig. 7c) and percentages of free alveolar space (Fig. 7d), we observed microscopically higher degree of inflammation in the lung of p55^∆NS^ mice than in WT mice (Fig. 7e).

**Figure 8:**
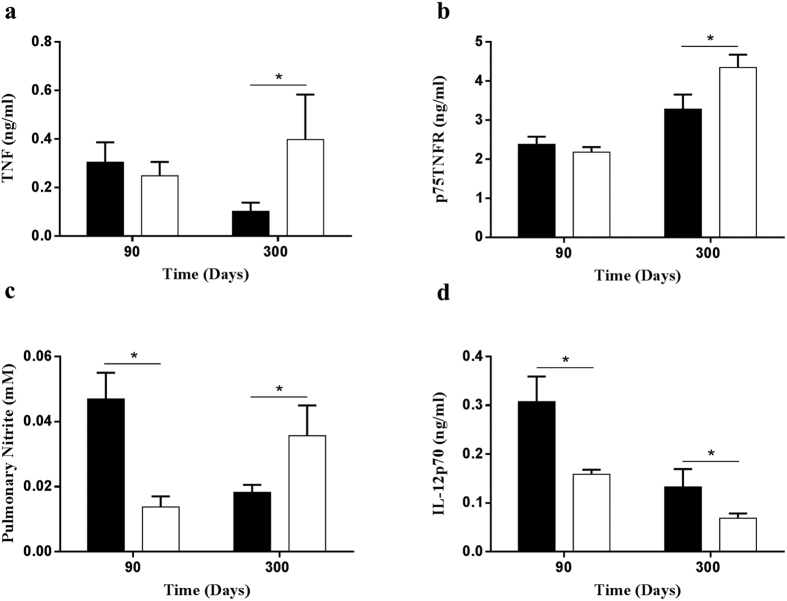
Cytokine productions in mice expressing non-sheddable form of p55TNFR during reactivation of latent infection. Lungs were isolated from WT mice (closed bars) and p55^∆NS^ mice (open bars), homogenized at day 90 and 300 post-infection, and the levels of TNF (**a**), p75TNFR (**b**) and IL12p70 (**d**) were quantified by ELISA and Nitrite (**c**) was quantified by Griess reagent. Results represent 1 of 3 similar experiments and data are expressed as mean ± SD of triplicate values. Statistical analysis was performed by ANOVA (*p < 0.05, **p < 0.01, ***p < 0.001).

To further assess the effects of persistent p55TNFR expression on p75TNFR levels and the cytokine productions during reactivation of latent M. tuberculosis infection, we compared the levels of TNF, p75TNFR, NO and IL-12p70 in the lungs of WT mice and p55^∆NS^ mice at 90 and 300 days post-infection. Both TNF and p75TNFR levels in p55^∆NS^ mice were similar to WT mice at day 90 but significantly increased at day 300 (p < 0.05) (Fig. 8a,b). Nitrite concentrations in p55^∆NS^ infected lungs were significantly lower at day 90 (p < 0.05) but higher at day 300 (p < 0.05) than the WT lungs (Fig. 8c), Pulmonary IL-12p70 concentrations were reduced significantly (p < 0.01) in p55^∆NS^ mice at both day 90 and 300 (Fig. 8d).

**Figure 9:**
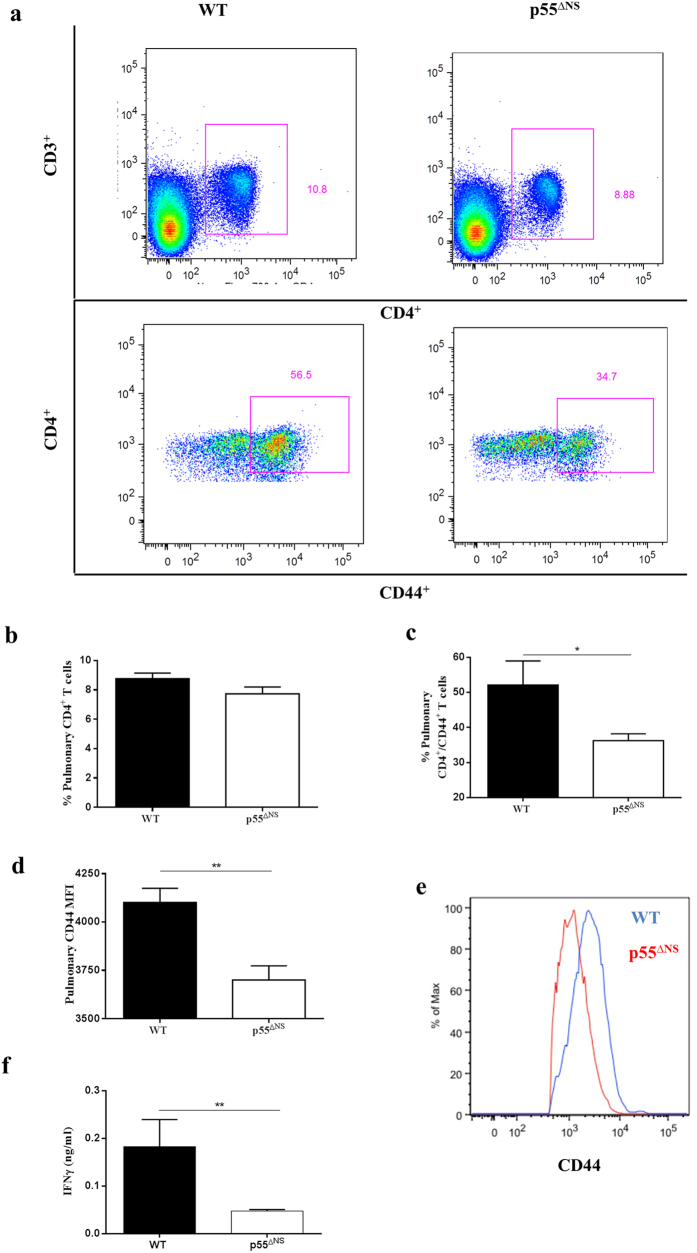
Reduced effector T cells in the lungs of latently *M. tuberculosis infected p55*^*∆NS*^ *mice*. WT mice (closed bars) and p55^∆NS^ mice (open bars) were infected by aerosol inhalation with 50–100 CFUs/mouse of *M. tuberculosis* H37Rv. Latency was achieved by treatment with RIF/INH (25 mg/kg) for 6 weeks commencing at day 28 post-infection. Single cell suspensions were generated from PBS-Heparin perfused lungs at day 90 post-infection, stained and analyzed for CD3, CD4, and CD44 expression by flow cytometry. Representative dot plots depicting flow cytometry analysis of CD4^+^ and CD4^+^/CD44^+^ cells (**a**) obtained from the infected lungs of WT and p55^∆NS^ mice. Percentages of pulmonary CD4^+^ cells (**b**) and CD4^+^CD44^+^ effector T cells (**c**) were determined. The MFI of CD44 gated on CD4^+^ T cells is depicted in (**d**) with a representative histogram overlay in E (IgG control – green, WT – red, p55^∆NS^ - blue). Data represent 1 of 2 experiments performed and results are expressed as mean ± SD of 5 mice/group. Statistical analysis was performed by ANOVA (*p < 0.05, **p < 0.01, ***p < 0.001).

We next assessed the quality of the T cell response generated during latent infection by analyzing the activation state of CD4^+^ T-cells in infected lungs after 6 weeks of rifampicin and isoniazid treatment, and a further 3 weeks of infection reactivation. While comparable frequencies of pulmonary CD4+ T cells were present between the two strains at 90 days post-infection (Fig. 9a,b), the frequencies of pulmonary CD4^+^CD44^+^ T cells were reduced in p55^∆NS^ mice (Fig. 9a,c). The MFI of cell surface CD44 gated on CD4^+^ T cells was also significantly lower in p55^∆NS^ mice compared to WT mice (Fig. 9d,e). Moreover, we observed significantly lower IFNγ concentrations in p55∆NS mice compared to WT mice (Fig. 9f).

Together the data indicates an inability of p55^∆NS^ mice to control reactivation of latent tuberculosis, which may be linked to the fact that persistent p55TNFR expression suppresses effector T cell activation during latent infection.

## Discussion

TNF-p55TNFR mediated signaling is a critical requirement for control and resolution of mycobacterial infection^21^,^22^,^28^,^29^. The widespread expression of TNFRs on different cell types and tissues^55^,^56^,^57^ indicates the spectrum breath of target cells with which TNF can interact and necessitates tight regulation around the TNF-TNFR signaling axis. TNF mediated activities are controlled by multiple regulatory mechanisms including proteolytic shedding of TNFRs from cell surfaces. The resultant soluble p55TNFR and p75 bind TNF, competing with cell surface receptors and limit TNF function in target cells^35^,^36^,^37^,^38^. We and others have shown TNFRp55 mediated TNF signaling to be essential to generate host immune protection, while shed, soluble p75TNFR has a critical role in regulating TNF bioavailability^15^,^16^. Clinically, increased soluble p55TNFR and p75TNFR has been associated with exacerbated *M. tuberculosis* disease severity^18^,^19^,^30^. In humans, specific missense mutations in the p55TNFR gene that cause defective receptor shedding strongly associate with autosomal dominant periodic fever syndromes known as TRAPS^44^, and are thought to be primarily caused by irregular innate immune function in these patients. The effects of persistent membrane TNFR expression, in the absence of the shed form, within disease settings are unknown.

In this study, we investigated the functional relevance of persistent p55TNFR cell surface expression and impaired p55TNFR shedding during infection with *M. tuberculosis* H37Rv using a mutant mouse strain (p55^∆NS^) with p55TNFR wild type receptor functionality but defective shedding capability^34^. We confirmed that p55TNFR and p75TNFR are released from the cell surface prior to and after macrophage activation with either LPS or viable *M. tuberculosis* H37Rv bacilli, in agreement with previous reports demonstrating that soluble TNFRs are generated after cell activation^58^,^59^,^60^. Moreover, our results show that both constitutive and *M. tuberculosis* induced shedding are inhibited in p55^∆NS^ derived cells, correlating with persistency of p55TNFR cell surface expression. Interference of the TNF-p55TNFR signaling pathway either by gene deletion of ligand or receptor, or by neutralizing antibodies leads to susceptibility to mycobacterial and other intracellular pathogens^21^,^29^,^61^,^62^,^63^. We hypothesized that persistent cell surface p55TNFR expression would result in enhanced TNF-p55TNFR interaction leading to improved disease outcome. In contrast, our data demonstrate that persistent cell surface p55TNFR expression does not confer a protective advantage against *M. tuberculosis* H37Rv infection in either acute or chronic infection. We have previously reported that treatment of WT dendritic cells with recombinant p75TNFR reduced bioactive TNF levels which resulted in impaired dendritic cell activation. In contrast treatment with anti-p75TNFR had the opposite effect with increased bioactive TNF translating to enhanced dendritic cell activation^16^. We would therefore postulate an enhanced protection in these p55^∆NS^ mice by blocking its p75TNFR to increase macrophage activation and killing ability. Although the p55^∆NS^ macrophages from our *in vitro* experiments exhibited better bactericidal potential, the p55^∆NS^ mice failed to demonstrate improved bacterial control, which was expected considering the similar *in vitro* bacilli killing abilities in WT and p55^∆NS^ macrophages. However caution must be exercised when directly comparing *in vitro* and *in vivo* data; the antigen presenting cells (APC’s) in the lungs are comprised of a more complex array of cells which include inflammatory monocytes, alveolar macrophages and dendritic cells. The alveolar macrophages are the key cells to first line defense against *M. tuberculosis* in the lung; however they are not the ideal cell type for our *in vitro* experiments to study bacilli killing. We used IFNγ activated BMDM’s resembling inflammatory monocytes, whose primary immune function is bacilli killing during infections.

We found that mice expressing the mutant persistent cell surface receptor succumbed to *M. tuberculosis* chronic infection earlier than the WT group with a significant increase in bacilli burdens. The p55^∆NS^ mice also demonstrated a loss of bacilli control resulting during latent infection. These data demonstrate that the host protection against tuberculosis infection was not improved during persistent p55TNR expression as initially hypothesized. These findings contrasted earlier studies which reported that challenging p55^∆NS^ mice with increasing doses of *L. monocytogenes* results in improved host resistance compared to control animals. Under such conditions sustained p55TNFR expression enhanced antibacterial host mechanisms and suggested that *L. monocytogenes* might evoke p55TNFR shedding as an immune escape mechanism^34^. This contrast highlights the differences in the pathogenesis and immunity of these two pathogens. For example, the functions of membrane TNF has been studied in both listeria and tuberculosis disease models. Using the mice with noncleavable membrane TNF (mem-TNF) Torres *et al*. showed partial protection against Listeria in a dosage dependent manner^64^. On the other hand, Fremond *et al*. reported that the mem-TNF mice were protected from the Mycobacterium tuberculosis acute infection but not the chronic infection^65^.

We investigated host immune responses in p55^∆NS^ mice to gain deeper understanding of immune function during *M. tuberculosis* infection. We found that pulmonary cellular recruitment did not increase over the course of infection in p55^∆NS^ mice relative to control animals. This outcome was interesting, as TNF is known to be a master orchestrator of inflammation. Closer inspection of the lung cell populations during early infection revealed a transient increase in the frequency of CD11b^+^/MHC-II^+^ expressing cells in mice persistently expressing p55TNFR, however these cells displayed a state of activation equivalent to WT control mice. Moreover, we found persistent p55TNFR expression associated with defective pulmonary IFNγ synthesis correlating with a decrease in CD4^+^ T cell activation despite normal pulmonary CD4^+^ T cell recruitment. Collectively, our findings suggest that persistent p55TNFR expression induces hypo-immune response during *M. tuberculosis*. This is in line with the fact that I T cell hybridoma clones exposed to chronic TNF stimulation down regulated TCRζ and TCR/CD3 complex cell surface expression, leading to T cell hyporesponsiveness reflected by a decrease in antigen-specific proliferation and suppression of cytokine responses^66^. The authors further demonstrated that the observed T cell hyporesponsiveness was mediated via p55TNFR^66^. Persistent p55TNFR signaling also led to down modulation of T cell responses in a model of chronic inflammation involving TCR transgenic mice^67^. Collectively the data supports a mechanism by which T cell activation is regulated in a p55TNFR dependent manner during chronic *M. tuberculosis* infection. This is likely indirect, as we showed recently that the p55TNFR pathway in T cells is dispensable for controlling *M. tuberculosis* infection^26^. However, given our previous published data in which we demonstrated an increased p75TNFR shedding in the absence of p55TNFR resulting in reduced bioactive TNF^16^, we hypothesized that a similar compensationary increase in p75TNFR in the non-sheddable p55TNFR mouse strain and subsequent reduced bioactive TNF levels may be the underlying mechanism driving the observed T cell hyporesponsiveness in the p55^∆NS^ mice. Indeed an analysis of soluble p75TNFR both *in vitro* in bone marrow derived macrophages as well as *in vivo* in BAL fluid samples, demonstrated an increased level of soluble p75TNFR in p55^∆NS^ mice compared to WT mice which we confirmed *in vivo* to induce impaired bioactive TNF concentrations. We have also previously shown that bioactive TNF signaling through p55TNFR in dendritic cells plays an important role in IL-12 dependent DC migration to the draining lymph node and subsequent *M. tuberculosis-*specific T cell activation^16^. It is therefore possible that disruption of p55TNFR shedding leads to a compensationary increase in soluble p75TNFR which reduces bioavailable TNF and impairs dendritic cell migration, all of which results in defective Th1 responses.

Our hypothesis of a potential defect in T cell responses during persistent p55TNFR expression was indeed validated when p55^∆NS^ mice displayed enhanced sensitivity to tuberculosis reactivation. The mechanism associated with defective T cell responses during chronic tuberculosis is unknown. Given the similar frequencies of T cell recruitment, a reduction in T cell proliferation is unlikely to be the cause of defective T cell responses. This opens the possibility to a potential role for regulatory T cells (Tregs) in the suppressed CD4^+^CD44^+^ T cell activation during persistent p55TNFR expression. Previous studies have indicated that neutralization of TNF during rheumatoid arthritis treatment elicited a significant population of Tregs. And the ablation of p55TNF in reactive arthritis mice resulted in a reduced Tregs number and activity indicate that TNF signaling through p55TNFR and p75TNFR is important to the regulation of Treg cell function^68^,^69^. Another possible mechanism associated with defective T cell responses might be that sustained p55TNFR signaling induces T cell exhaustion. Recently, Beyer *et al*., reported reversal of T cell exhaustion through TNF neutralization which reconstituted specific immunity against chronic LCMV infection^70^. Interestingly, selective TNF receptor expression on T cells was shown to be responsible for T cell exhaustion.

In conclusion, our results demonstrate that while sustained p55TNFR expression mediated normal acute control of acute *M. tuberculosis* infection, it was associated with suboptimal T cell responses during chronic infection and promoted tuberculosis reactivation.

## Methods

### Mice

C57BL/6 wild type (WT) and p55^∆NS^ mice^34^ were maintained and housed in individually ventilated cages under specific pathogen free conditions in the Research Animal Facility at the University of Cape Town (South Africa). For all the experiments, age matched mice were used.

### Ethics statement

All experiments and protocols were approved and performed in accordance with the guidelines of the Research Ethics Committee of the University of Cape Town, South Africa and complied with South African regulations as stipulated in *The South African National Standard 10386-The care and use of animals for scientific purposes*.

### Mycobacteria and infection

*M. tuberculosis* H37Rv was grown in Middlebrook 7H9 broth (Becton, Dickinson and Company, Le Pont de Claix, France) supplemented with 10% Middlebrook OADC enrichment medium (Life Technologies, Gaitherburg, MD), 0.5% glycerol and 0.05% Tween 80 at 37 °C until log phase. H37Rv-GFP (kindly provided by Joel Ernst, University of San Fransciso, California) was grown similarly with 25 mg/L kanamycin in the broth. Prior to use, mycobacterial aliquots were passed 30× through a 29.5G needle to minimize bacterial clumping. Pulmonary infection at a dose of 50–100 live *M. tuberculosis* H37Rv bacilli was performed using a Glas-Col Inhalation Exposure System, Model A4224. Inoculum dosage was confirmed 24 h post-infection by determining the bacilli burden in the lungs of infected mice. To establish a latent infection, mice were treated with 25 mg/Kg INH-RIF for 6 weeks commencing at day 28 post- infection, which resulted in undetectable levels of bacilli. For disease reactivation, chemotherapy was withdrawn and bacilli burdens determined at day 90, 180 and 300 days post infection. For colony enumeration organs were weighed and homogenized in 0.04% Tween 80/PBS. Tenfold serial dilutions of organ homogenates were plated in duplicates on Middlebrook 7H10 (Becton, Dickinson and Company) agar plates containing 10% OADC (Life Technologies, Gaitherburg, MD) and incubated at 37 °C for 19–21 days.

### Lung Morphology

Lung weights were measured for use as a surrogate marker of inflammation, and thereafter fixed in 10% formalin and paraffin-embedded. Tissues were sectioned at 2–3 μm and stained with haematoxylin and eosin (H&E), then analysed using a Nikon 90i Eclipse microscope. The percentage of free alveolar space was calculated based on the area measurement using the microscope imaging software NIS-Elements, Nikon to analyze the open space in the whole lung section.

### Cytokine ELISA

Whole lungs were homogenized in 1 ml 0.04% Tween 80 saline containing protease inhibitor (Sigma) and the supernatants, aliquoted and frozen at −80 °C. Cytokine concentrations were measured in organ homogenates or supernatants from cultured cells using commercially available ELISA reagents for TNF, p55TNFR, p75TNFR, IFNγ, and IL-12p70, (R&D Systems, Germany and BD Pharmingen, San Diego), according to the manufacturer’s instructions.

### Lung single cell preparation and Flow cytometry

Lungs from infected mice were perfused by injecting 5 ml cold PBS containing 20 U/ml heparin (Bodene (PTY) Limited, RSA) in the right ventricle of the heart. Lungs were removed, sectioned and incubated in PBS containing 50 U/ml collagenase 1 (Worthington Biomedical Coorporation, Lakewood, NJ) and 13 μg/ml DNAse 1 (Boeringer-Mannheim, Germany) at 37 °C for 90 min with rotation. For single cell suspension, lung tissue was passed through a 70 μm nylon cell strainer (Beckton and Dickinson), washed 2× with PBS and viable cell numbers were determined by counting in the presence of trypan blue.

Isolated lung cells from infected mice were stained with the following antibodies: anti-CD3 BV421 (145-2C11), anti-CD4-FITC (H129.19), anti-CD4 Alexo-fluor 700 (RM4-5), anti-CD44-PE (IM7), anti-CD86-PE (GL1), anti-CD80-PE (16-10A1), anti-I-A/I-E-PE (M5/14.15.2), anti-CD11b-FITC. All the antibodies were purchased from Pharmingen and used at 2 μg/ml/10^6^ cells. Cells were fixed in 4% paraformaldehyde and were acquired using a FACS Calibur or BD LSR Fortessa (Beckton and Dickinson) and analyzed using FlowJo 7.5 software (Tree star, Ashland, OR, USA).

### Peritoneal macrophages

Mice were injected with 3% thioglycollate (Difco, St. Louis, USA). Five days later, peritoneal exudate cells were isolated from the peritoneal cavity by washing with ice-cold RPMI (Sigma, Germany) supplemented with 10% FCS (Gibco, Invitrogen Corporation, Germany). Cells were cultured overnight at 37 °C and 5% CO_2_ incubator. Adherent monolayer cells were used as peritoneal macrophages and were cultured at 5 × 10^5^ cells/ml in RPMI supplemented with 10% FCS and stimulated for 20 min with either 100 ng/ml LPS (*E. coli*, serotype O111:B4, Sigma) or *M. tuberculosis* H37Rv (MOI 2:1).

### Primary macrophage cultures

Bone marrow cells were isolated from femurs of 6 to 8 weeks old naive mice and were cultivated on 90 mm Sterilin plates (Bibby Sterilin, UK) at 2 × 10^6^ cells/ml for 7 days in RPMI (Sigma, Germany) supplemented with 2 mM L-glutamine (Gibco, Invitrogen Corporation, Germany), 0.2 μM 2-ME (Sigma, St. Louis, USA), 20% horse serum (Gibco, Invitrogen Corporation, Germany) and 30% L929 cell-conditioned medium at 37 °C and 5% CO_2_. Confluent cells were harvested and seeded at 5 × 10^5^ cells/ml in 96 well tissue culture plates (Nunclon, Denmark) and incubated for 24 h at 37 °C and 5% CO_2_ to allow for cell adherence. Cells were then stimulated with either 100 ng/ml LPS (*E. coli*, serotype O111:B4, Sigma) or live *M. tuberculosis* H37Rv bacteria (MOI 2:1) for 90 min, 150 min, 300 min, 24 h, and 48 h. Supernatants were collected and analysed for cytokine content using ELISA and nitrite concentration using Griess reagent (3% phosphoric acid, 1% p-aminobenzene-sulphonamide, 1% n-naphthylenediamide) as described^71^.

To determine bactericidal capacity to kill *M. tuberculosis* bacilli, BMDMs were infected with H37Rv-GFP at 2:1 MOI for 4 to 120 hours. The amount of intracellular bacilli was determined by lysing the macrophages and measuring the relative fluorescent unit (RFU) using Modulus microplate fluorometer (Turner Biosystems Inc.).

### BAL collection

Mice were euthanized to reveal the trachea. BAL fluid was collected by 2 lavages in which an 18-gauge cannula was inserted into the trachea and aspirated 10 times with 300 μl sterile PBS. The BAL fluid was aliquoted and stored at −80 °C for cytokine analysis. BAL cells were collected by 3 lavages with 700 μl sterile PBS each. Cells were kept on ice for flow cytometric analysis.

### Bioactive TNF assay

Bioactive TNF was determined as previously described^16^, using TNF sensitive fibroblast cell line WEHI 164, clone 13 (Walter and Eliza Hall Institute). WEHI cells were cultured in RPMI (Sigma, Germany) containing 10% FCS (Gibco, Invitrogen Corporation, Germany), 10 U/ml penicillin (Gibco, Germany), 10 μg/ml streptomycin (Gibco, Invitrogen Corporation, Germany) and 0.5× amino acid supplement (MEM Amino acids without L-glutamine, Gibco, Germany) at 37 °C with 5% CO_2_. Confluent cells were reseeded at 2 × 10^5^ cells/ml in 96 well tissue culture plates (Nunclon, Denmark). TNF standards (recombinant mouse TNF, BD PharMingen, San Diego) in 2 fold serial dilutions or samples were added to WEHI cells and incubated for 18 h at 37 °C with 5% CO_2_. MTT solution (2 mg/ml, Sigma, Germany) was then added and incubated for a further 2 h, after which, the supernatants were aspirated and 50 μl DMSO was added and samples were read at 570 nm using VERSAmax Tunable Microplate Reader (Molecular devices Coorporation, California, USA). Data was analyzed using SoftMax Pro (Molecular devices Coorporation, California, USA).

### Statistical Analysis

Statistical analysis was performed using Graphpad Prism 6 by ANOVA. For mortality studies, analysis was performed using the logrank test. For all tests, a p value of <0.05 was considered significant.

## Additional Information

**How to cite this article**: Dambuza, I. M. *et al*. Persistent p55TNFR expression impairs T cell responses during chronic tuberculosis and promotes reactivation. *Sci. Rep.* **6**, 39499; doi: 10.1038/srep39499 (2016).

**Publisher's note:** Springer Nature remains neutral with regard to jurisdictional claims in published maps and institutional affiliations.

## Supplementary Material

Supplementary Information
