# Supplementary material for: Persistent p55TNFR expression impairs T cell responses during chronic tuberculosis and promotes reactivation
Source: Sci Rep. 2016 Dec 20;6:39499. doi: 10.1038/srep39499 (PMC5171238; doi:10.1038/srep39499)
Supplement: Supplementary Information [file srep39499-s1.pdf]

Persistent p55TNFR expression impairs T cell responses during chronic tuberculosis and promotes reactivation.

[*1#]Ivy M. Dambuza, *[1]Roanne Keeton, [1]Nai-Jen Hsu, [1#]Nasiema Allie, [2]Valerie J.F. Quesniaux, [2]Bernhard Ryffel, [1,3,4]Muazzam Jacobs.

[1] Division of Immunology, Department of Pathology and Institute of Infectious Disease and Molecular Medicine, Faculty of Health Sciences, University of Cape Town, South Africa

[2] CNRS UMR7355, Experimental and Molecular Immunology and Neurogenetics, 45071 Orleans, France

[3] National Health Laboratory Service, South Africa

[4] South African Medical Research Council

** Equal contribution*

[#]Current Address:

Ivy Dambuza: Institute of Medical Sciences, University of Aberdeen, Foresterhill, Aberdeen AB25 2ZD, UK.

Nasiema Allie: MRC Centre for Tuberculosis Research, Division of Molecular Biology and Human Genetics, Department of Biomedical Sciences, University of Stellenbosch

Corresponding Author

Muazzam Jacobs

Division of Immunology, Department of Pathology and

Institute of Infectious Disease and Molecular Medicine,

Health Sciences Faculty

University of Cape Town / National Health Laboratory Service

Observatory 7925,

South Africa.

Email: muazzam.jacobs@uct.ac.za

Tel: +27 21 406 6078

Running Title:

p55TNFR and Tuberculosis

Keywords

tumour necrosis factor, *Mycobacterium tuberculosis*, T cell, macrophage, TNF receptor, p55TNFR

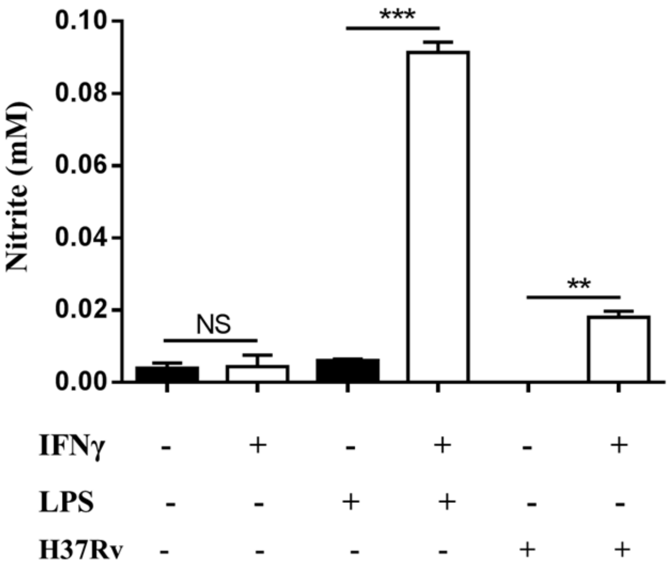

Supplementary Figure 1. Enhanced Nitric Oxide production in macrophages prestimulated with IFN$_\gamma$

Bone marrow derived macrophages isolated from WT mice were untreated (closed bars) or prestimulated (open bars) with 100 U/ml IFN$_\gamma$ for 24 h prior stimulation with control medium or 100 ng/ml LPS or *M. tuberculosis* H37Rv (MOI = 2:1). Results represent 1 of 3 similar experiments and data are expressed as mean ± SD of triplicate values. Statistical analysis was performed by ANOVA (* p<0.05, **p<0.01, ***p<0.001).

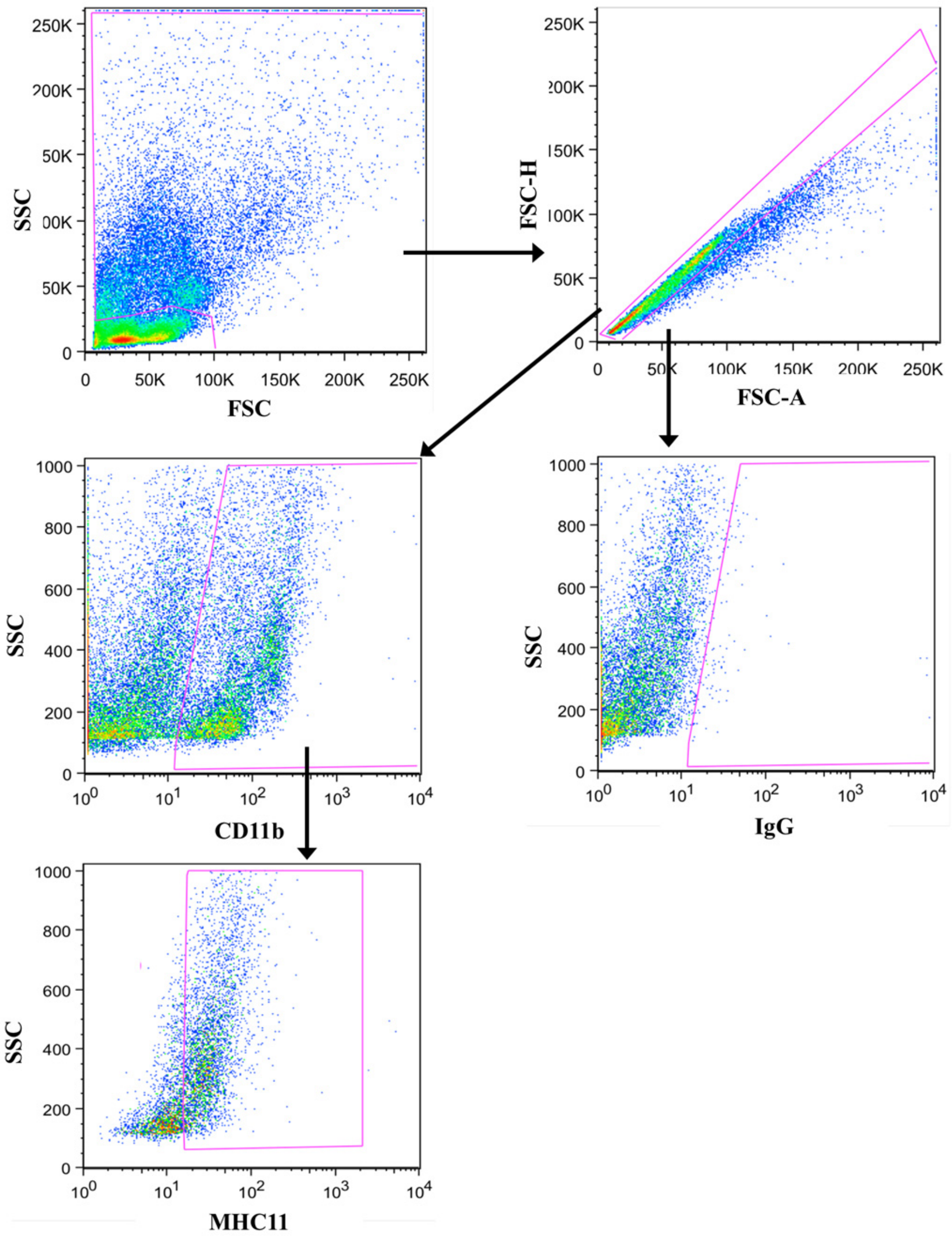

Supplementary Figure 2. Flow cytometry analysis of pulmonary CD11b $^{+}$ ***cells.***

Representative gating strategy to identify CD11b^{+} cells obtained from the mouse lungs.

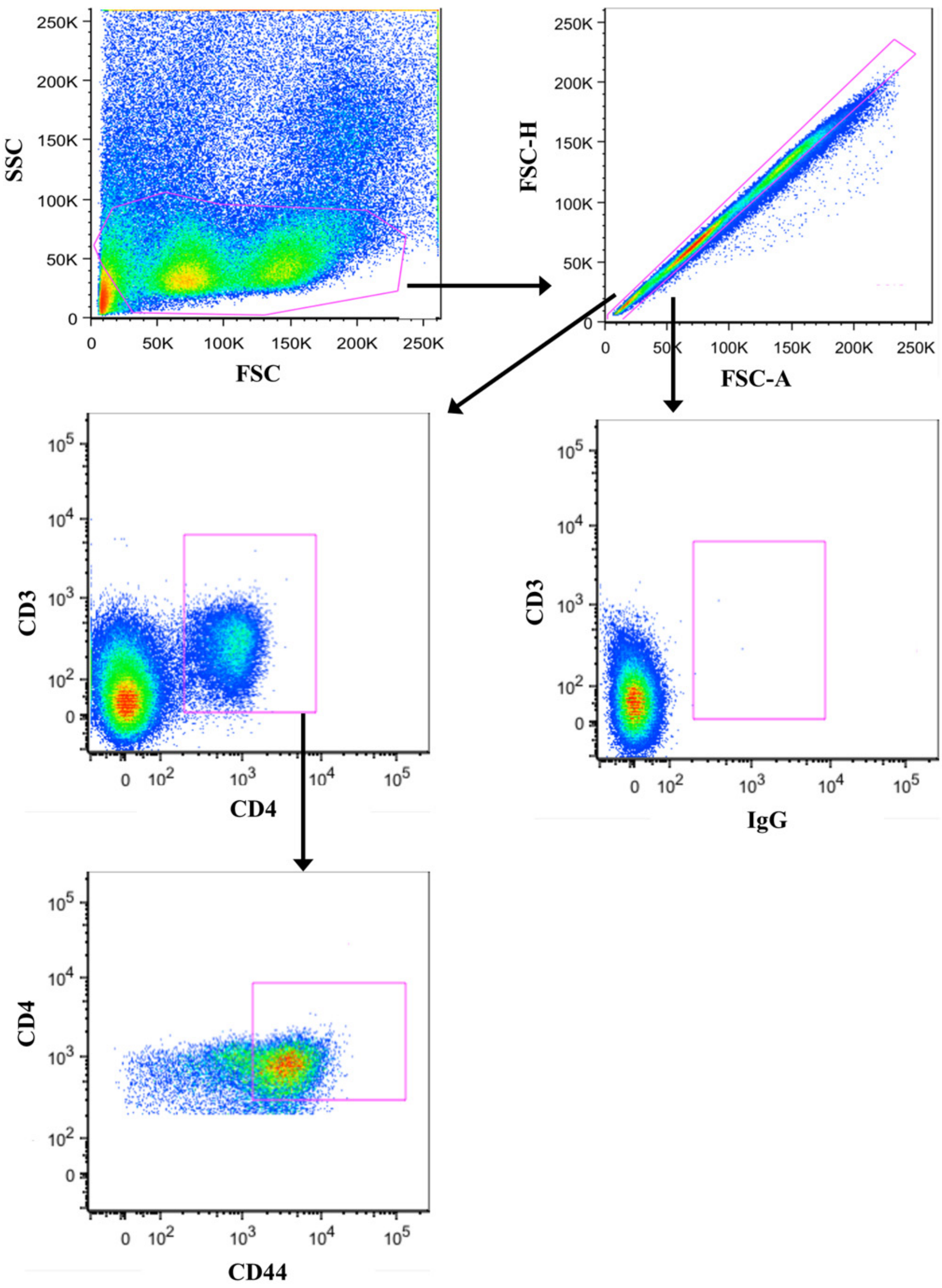

Supplementary Figure 3 Flow cytometry analysis of pulmonary CD4⁺ and CD44⁺ cells.

Representative gating strategy to identify $CD4^+CD44^+$ cells obtained from the mouse lungs.
